# 2280 Subjective, physiological activation and habituation, and response to written trauma narrative exposure

**DOI:** 10.1017/cts.2018.182

**Published:** 2018-11-21

**Authors:** Mary K. Howell, Jennifer R. Myers, Thomas A. Mellman

**Affiliations:** 1 Georgetown – Howard Universities; 2 Walter Reed Army Medical Center; 3 Howard University

## Abstract

OBJECTIVES/SPECIFIC AIMS: Emotional processing theory and some observations suggest that activation of subjective and physiological distress during therapeutic exposure and habituation across exposure sessions are key to improvement. This study sought to determine whether initial subjective and physiological activation and between-session habituation would predict PTSD symptom reduction after a series of written trauma narrative exposure sessions. METHODS/STUDY POPULATION: In total, 29 urban-residing African-American participants with PTSD participated in four 30-minute writing sessions. Writing sessions 1 and 2 were 12 hours apart and session 3 and 4 were performed 1 week later, also 12 hours apart. PTSD symptoms were measured at baseline, after session 2, and 1 week after all 4 writing sessions with the Clinician Administered PTSD Scale. During each session, Subjective Units of Distress Scores (SUDS) were assessed 4 times and heart rate was measured continuously. RESULTS/ANTICIPATED RESULTS: Participants exhibited PTSD symptom improvement and habituation of subjective distress, but not physiological arousal, across writing sessions. First session baseline-corrected SUDS maximum and SUDS decrease from the initial to the final writing session were both positively associated with symptom improvement. DISCUSSION/SIGNIFICANCE OF IMPACT: Increased subjective, but not physiological, distress in the first exposure session and diminished subjective distress across sessions may be a helpful marker of emotional processing for clinicians and predictor of symptom improvement after written trauma narrative exposure.

